# Reinforcement learning evaluation of treatment policies for patients with hepatitis C virus

**DOI:** 10.1186/s12911-022-01789-7

**Published:** 2022-03-11

**Authors:** Brandon Oselio, Amit G. Singal, Xuefei Zhang, Tony Van, Boang Liu, Ji Zhu, Akbar K. Waljee

**Affiliations:** 1 Department of Biostatistics, University of Michigan, Ann Arbor, MI USA; 2 Department of Internal Medicine, Division of Digestive and Liver Diseases, UT Southwestern Medical Center, Dallas, TX USA; 3 Department of Statistics, University of Michigan, Ann Arbor, MI USA; 4 Health Services Research and Development Center of Clinical Management Research, VA Ann Arbor Healthcare System, 2215 Fuller Road, Gastroenterology 111D, Ann Arbor, MI 48105 USA; 5 Michigan Integrated Center for Health Analytics and Medical Prediction (MiCHAMP), Ann Arbor, MI USA; 6 Department of Internal Medicine, Division of Gastroenterology and Hepatology, Michigan Medicine, Ann Arbor, MI USA; 7 Googleplex, 1600 Amphitheatre Parkway, Mountainview, CA USA

**Keywords:** Hepatology, Prediction modeling, Cirrhosis, Reinforcement learning, Machine learning, Treatment policy, Risk-based treatment

## Abstract

**Background:**

Evaluation of new treatment policies is often costly and challenging in complex conditions, such as hepatitis C virus (HCV) treatment, or in limited-resource settings. We sought to identify hypothetical policies for HCV treatment that could best balance the prevention of cirrhosis while preserving resources (financial or otherwise).

**Methods:**

The cohort consisted of 3792 HCV-infected patients without a history of cirrhosis or hepatocellular carcinoma at baseline from the national Veterans Health Administration from 2015 to 2019. To estimate the efficacy of hypothetical treatment policies, we utilized historical data and reinforcement learning to allow for greater flexibility when constructing new HCV treatment strategies. We tested and compared four new treatment policies: a simple stepwise policy based on Aspartate Aminotransferase to Platelet Ratio Index (APRI), a logistic regression based on APRI, a logistic regression on multiple longitudinal and demographic indicators that were prespecified for clinical significance, and a treatment policy based on a risk model developed for HCV infection.

**Results:**

The risk-based hypothetical treatment policy achieved the lowest overall risk with a score of 0.016 (90% CI 0.016, 0.019) while treating the most high-risk (346.4 ± 1.4) and the fewest low-risk (361.0 ± 20.1) patients. Compared to hypothetical treatment policies that treated approximately the same number of patients (1843.7 vs. 1914.4 patients), the risk-based policy had more untreated time per patient (7968.4 vs. 7742.9 patient visits), signaling cost reduction for the healthcare system.

**Conclusions:**

Off-policy evaluation strategies are useful to evaluate hypothetical treatment policies without implementation. If a quality risk model is available, risk-based treatment strategies can reduce overall risk and prioritize patients while reducing healthcare system costs.

**Supplementary Information:**

The online version contains supplementary material available at 10.1186/s12911-022-01789-7.

## Background

Health system stressors occur in multiple medical contexts and can be exacerbated by limited resources (e.g., limited capacity or budget). This issue has become particularly apparent during the COVID-19 pandemic when resources such as personal protective equipment, intensive care unit capacity, and treatments have been in high demand but under limited supply [1–3]. During these situations, health systems must determine the most efficient and effective way to allocate scarce resources within the constraints of their health system, often to large populations of patients [3]. Some health systems take a first-come-first-serve approach, whereas others prioritize patients according to their risk of disease progression or complications. Allocation of care fairly and equitably is essential, particularly considering historic inequities with high barriers to care and worse health outcomes among racial and ethnic minorities and patients of low socioeconomic status. These policies have far-reaching effects and must be guided by the medical ethics [3–6]. Unfortunately, it is difficult to evaluate various approaches until after implementation. Although simulation modeling can be used to evaluate theoretical decision paths in advance, they are based on assumptions that may fail to provide unbiased evaluations of the hypothetical treatment policy [7]. These issues highlight a need for better methods to evaluate proposed policies before clinical implementation.

Herein, we used hepatitis C virus (HCV) treatment with direct-acting antivirals (DAA) as a case study on which to develop a reinforcement learning approach to evaluate proposed treatment policies before implementation using historical data. Reinforcement learning provides a framework to utilize data that is longitudinal in nature and contains feedback from decisions made over time, such as assessment of a patient’s health status and decision to start treatment and thus evaluate new treatment strategies or policies in medicine [8–14]. HCV is a valuable case study as it has traditionally been one of the most common risk factors for cirrhosis and liver-related mortality in the United States and Europe. The availability of DAA therapies in 2015 offered a cure for HCV and has helped mitigate HCV-related morbidity and mortality over the last decade. If patients are treated early before the onset of cirrhosis, HCV therapy can halt disease progression and significantly reduce the risk of cirrhosis and liver-related mortality. Patients treated after the onset of cirrhosis have improved quality of life and prognosis. However, they have a persistent risk of hepatocellular carcinoma and liver-related complications, warranting continued observation.

Despite the documented benefits of early treatment of HCV, the initial high cost of DAAs resulted in many payers limiting access to treatment. Some of these policies included restricted access to DAAs based on (1) fibrosis stage (e.g., presence of significant fibrosis or cirrhosis); (2) sobriety from alcohol and illicit substances; or (3) prescriber specialty. This was particularly true for Medicaid plans, which are responsible for covering low socioeconomic status groups and often racial and ethnic minority communities, thus exacerbating disparities in access and care—violating the ethical principle in equal access to care [4]. These policies led to institutional, geographic, and temporal variation in HCV treatment policies, including who is eligible and/or prioritized for treatment, thus creating historical treatment data and a natural case study to develop an approach to evaluate proposed treatment policies before implementation.

The purpose of this study was to evaluate hypothetical policies for HCV treatment before clinical implementation using techniques from reinforcement learning, leveraging historical data collected under an existing treatment policy. With this historical data, we can evaluate new hypothetical treatment policies under the paradigm of reinforcement learning by comparing them with the resulting rewards (lower risk) of the existing treatment policy. In this setting, we wish to evaluate hypothetical treatment policies that could potentially replace the standard-of-care policy. With HCV as our case study, we used a previously published risk prediction model [15] to measure a patient's risk over time. This published model was the basis for patients' risk estimates and the hypothetical risk-based treatment policy. This risk estimates combined with the treatment decisions made for each patient and associated longitudinal and demographic variables allowed us to compare additional hypothetical treatment policies for HCV treatment allocation.

## Methods

### Data collection and study population

The cohort was collected from the Veterans Health Administration (VHA) Corporate Data Warehouse, an electronic repository of clinical and demographic data for Veterans served by the VHA health care system. All patients with a history of HCV (defined by the presence of at least one positive HCV RNA during the study period) were identified from January 2015 to January 2016, with follow-up through 2019. The original cohort study was obtained from a previous publication whose original study date was from January 2000 to January 2016 and had the following exclusions: patients with less than two AST-to-platelet ratio index (APRI) scores, patients with a history of cirrhosis or hepatocellular carcinoma at baseline, and those with baseline APRI > 2.0, and finally excluded those patients who received antiviral treatment regimens but lacked RNA tests to document whether sustained virologic response (SVR) was achieved. The resulting dataset consisted of 169,339 patients. From this dataset and given that the focus of our study was DAA receipt, we excluded patients seen before January 2015 (n = 164,835) and those that were only treated with older non-DAA interferon-based regimens (n = 34). We also excluded patients that were treated with DAAs but did not achieve sustained virologic response (SVR, i.e., HCV cure) (n = 397); and patients that needed to be treated with more than one antiviral regimen (n = 138); finally, we excluded any patients whose treatment occurred before study enrollment (n = 143). This led to 3792 patients in the final dataset.

### Study variables

Predictors of interest were selected a priori based on prior work, [15, 16] biological plausibility [15–17], and clinician input. Demographic variables included age at cohort entry, sex, race, and ethnicity. SVR, i.e., HCV cure, was modeled as a step function of time whereby the variable remains 0 until SVR is achieved, at which point it becomes 1. Laboratory variables included aspartate aminotransferase (AST) ratio, alanine aminotransferase (ALT) ratio, AST/ALT ratio, albumin, total bilirubin, creatinine, blood urea nitrogen, glucose, hemoglobin, platelet count, white blood cell count, sodium, potassium, and chloride. We used all available laboratory measurements for each patient. Patients with more than one measurement for a particular variable on a single day were averaged for that day. For each patient, treatment information is also available (if they were treated), including the type of drug, treatment date, and treatment length. This allows us to create longitudinal patient trajectories and treatment decisions over time.

### Reinforcement learning algorithm approach for treatment policies

Reinforcement learning is an area of machine learning that studies how actions are taken over time affect current and downstream outcomes [8–14, 18]. An example is a physician who is considering different treatment options for a specific condition, which may require updating based on a patient’s response and any adverse events. Reinforcement learning can help determine a policy of action best for patients on average. This differs from traditional machine learning, where temporality is not taken into effect, and decisions cannot vary over time.

**Fig. 1:**
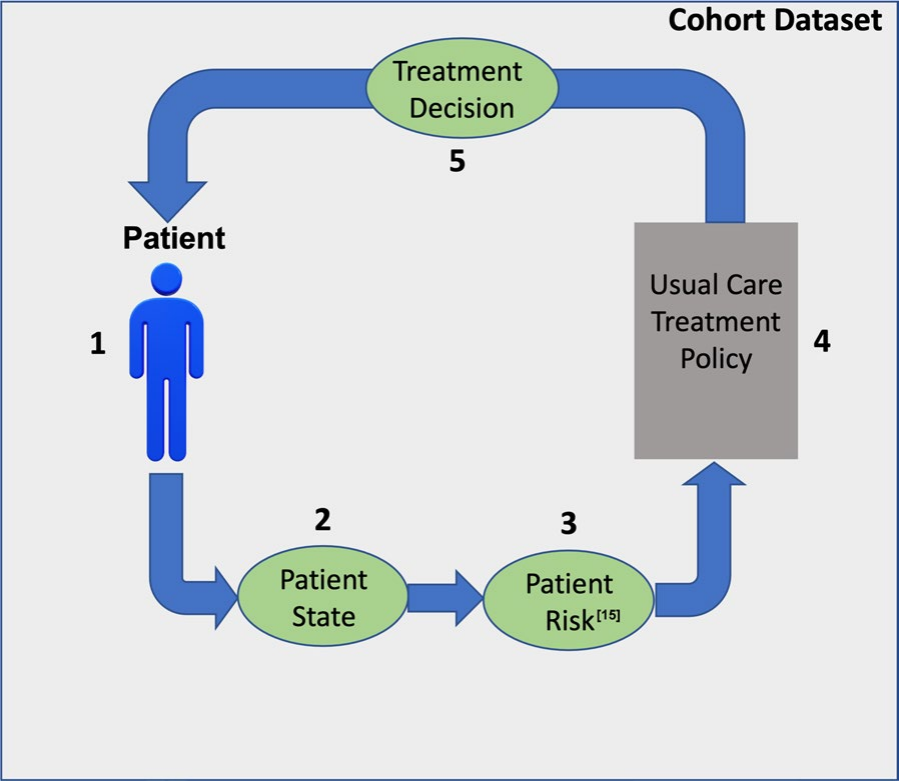
Modeling approach for reinforcement learning and off-policy evaluation. The historical cohort dataset consists of patients (1), whose state, i.e., longitudinal and demographic information is measured (2). Given these measurements, the risk to the patient progressing to cirrhosis is then evaluated (3). Finally, following the usual care treatment policy (4), a clinician makes a treatment decision (5) for the patient. The cycle then continues until the patient no longer returns for follow-up or the follow-up period concludes

Reinforcement learning can be used to both evaluate sequential decision-making and identify and evaluate new policies in medicine. In the original cohort data (Fig. 1), treatment decisions are made in a sequential context: the patient is evaluated, and information, or the patient’s state, is collected. The clinician then evaluates this state and makes a treatment decision. This cycle then restarts as the patient’s state is measured again, and the clinician updates the treatment decision based on this new information. The process continues for a series of time points until the patient does not return for more visits or the end of the follow-up period is reached. In this setting, we wish to evaluate hypothetical treatment policies that could potentially replace the standard-of-care policy; ideally, a hypothetical treatment policy would have a lower overall risk to patients. Reinforcement learning offers a framework to encode this process quantitatively.

In general, this requires a multi-step approach: (1) A feature representation is created for the states, actions, and risks for each patient in the dataset, (2) an off-policy evaluation method is constructed as a way to compare hypothetical treatment policies, and (3) hypothetical clinical policies are tested utilizing the off-policy evaluation method.

1. Feature Representation for States, Actions, and Risks

A set of longitudinal states, actions, and risks is derived from the cohort data for each patient. A measurement time point for each patient is defined as a day of record for the patient in the cohort data, i.e., if the patient has a recorded longitudinal measurement on that day.

**States**: The state for each patient at each time point is a vector of the following variables: sodium, creatinine, chloride, total protein count, alkaline phosphatase, APRI, potassium, glucose, platelet count, AST ratio, INR, white blood count, bilirubin, albumin, ALT ratio, AST/ALT ratio, FIB4 score, SVR status, demographic group (Hispanic, White, or Other), and sex. For the demographic information and sex, the variable is coded as 1 for True and 0 for False (demographic is split into three separate variables). Those values are constant over time for the patient. For missing values, the last known value carried forward is used. When a measurement is missing for the patient at all measurement time points, the overall median of that variable across all patients is used.

**Actions**: For the HCV cohort, we consider a binary treatment decision. The action variable at each time point is 1 if the treatment is currently occurring. If there is no measurement time point on the first day of treatment, we define the action at the previous measurement time point as 1. This is done so that the time at which a treatment decision is made is correctly recorded, i.e., during a defined measurement time point.

**Risks**: The treatment decision under consideration is whether or not to treat with a DAA regimen. The patient’s risk is defined as the estimated risk of cirrhosis after 1-year. We calculated patient risk at each measurement time point using a predefined model from Beste et al. [15], which used a multivariate time-varying Cox process to predict the risk of cirrhosis within 1-year from the current time point and was specifically adapted to use longitudinal lab data and to account for treatment and SVR status. The coefficients of the time-varying Cox Model were previously published in Beste et al. (2020) and found in Additional file 1: Table S1 [15].

2. Off-Policy Evaluation

Off-policy evaluation in this paper is based on the reinforcement learning framework to evaluate the hypothetical scenarios rather than learn a new policy using historical data [19–22]. To adequately describe this technique, we first introduce some notation. As described in the previous section, we obtain for each patient a sequence of triplets consisting of actions${a}_{i}$, states${s}_{i}$, and risks${r}_{i}$, $H=[\left({a}_{1},{s}_{1},{r}_{1}\right), \ldots \left({a}_{T}, {s}_{T}, {r}_{T}\right)]$. We mathematically define a treatment policy $\uppi$ as a probability distribution over the possible action space, given a particular state, so that in our case $\pi \left(1 \right| s)$ is the probability of treatment with a DAA given the patient states, and $\pi \left(0 \right| s)=1 -\pi \left(1 \right| s)$. Given this treatment policy $\uppi$, we are interested in estimating the overall risk to patients over time: where $\gamma \in [0, 1)$ is a discount parameter that encodes how much we care about past risk as opposed to current risk of the patient, and ${\mathrm{r}}_{\mathrm{t}}$ are the risks observed when implementing the treatment policy $\uppi$ at time t. If enough data is collected under the policy of interest, then it is straightforward to estimate Eq. 1 by simply using the empirical weighted average of the resulting risks for each patient. However, as we wish to use historical cohort data to evaluate $\uppi ,$ we resort to a different approach. In particular, we utilize a statistical technique called importance sampling, which reweights the data to estimate risk under the policy of interest (6).

1 $${\text{Risk}}(\pi ) = {\mathbb{E}}\left[ {\sum\limits_{t = 0}^{T} {\gamma^{t} r_{t} } } \right]$$

Let ${\pi }_{e}$ be the treatment policy we wish to evaluate. Similarly, we define ${\pi }_{b}$ as the baseline treatment policy under Eq. 2, which the data was collected. Then, for each patient i and each time t, we can define the importance weight: where $({a}_{j}^{\left(i\right)}, {s}_{j}^{(i)})$ is the action-state pair for patient i at time j

2 $$\rho_{t}^{(i)} = \prod\limits_{j = 0}^{t} {\frac{{\pi_{e} (a_{j}^{(i)} |s_{j}^{(i)} )}}{{\pi_{b} (a_{j}^{(i)} |s_{j}^{(i)} )}}}$$

**Estimation of** $\boldsymbol{\uppi }_{\mathbf{b}}$: To calculate the importance weights, it is necessary to access both ${\pi }_{e}$ and ${\uppi }_{\mathrm{b}}$ for all possible actions and states. Since ${\uppi }_{\mathrm{e}}$ is the hypothetical evaluation policy, for every possible state $\mathrm{s}$ we know the probability of a treatment decision ${\uppi }_{\mathrm{e}}(\mathrm{s})$. For ${\uppi }_{\mathrm{b}}$, however, these probabilities must be estimated from the data.

Since the treatment decision is binary, estimating the baseline treatment policy ${\uppi }_{\mathrm{b}}$ can be done by fitting a probabilistic classifier to the actions and states and using the posterior probabilities of an action $\mathrm{a}$ (output of the classifier) given the state $\mathrm{s}$ (input to the classifier). Logistic regression was chosen for this task as it has worked well and was a reasonable choice. To account for the fact that DAA treatment regimens are fixed and would not change with implementing a new treatment policy, the learning of ${\uppi }_{\mathrm{b}}$ was split into two phases: pre-treatment ${\uppi }_{\mathrm{b}}^{\mathrm{pre}}$ and post-treatment ${\uppi }_{\mathrm{b}}^{\mathrm{post}}$. All action-state pairs from all patients that occurred before treatment or were on the treatment decision day were used for the pre-treatment phase.

For the post-treatment phase, we set ${\pi }_{b}^{post}\left(a \right| s)={\pi }_{e}^{post}\left(a \right| s)$ for all hypothetical treatment policies ${\uppi }_{\mathrm{e}}$ and for all possible treatment decisions and states. Note that, regardless of the estimated post-treatment policy${\pi }_{b}^{post}\left(a \right| s)$, the importance weight ${\rho }_{t}^{(i)}={\rho }_{{t}^{*}}^{(i)}$ for any $t\ge {t}^{*}$ , where ${\mathrm{t}}^{*}$ is the initial treatment time; it is, therefore, unnecessary to estimate ${\uppi }_{\mathrm{b}}^{\mathrm{post}}\left(\mathrm{a }\right|\mathrm{ s})$ directly.

After estimating the baseline policy ${\uppi }_{\mathrm{b}}$ and calculating the importance weights for each patient, a weighted estimator can then assess the overall average risk of the new treatment policies. For off-policy evaluation, we use a variant of the per-horizon weighted importance sampler found in Doroudi et al. & Raghu et al. [19, 21] and shown in Eq. 3.

3 $$\widehat{{V_{PHWIS} }} = \mathop \sum \limits_{l \in L} W_{l} \frac{1}{{\mathop \sum \nolimits_{{\tau_{i} | T_{i} = l}} \rho_{{T_{i} }}^{\left( i \right)} }} \mathop \sum \limits_{{\tau_{i} | T_{i} = l}} \rho_{{T_{i} }} \mathop \sum \limits_{t = 0}^{{T_{i} - 1}} \gamma^{t} r_{t}^{\left( i \right)}$$

γ is a discount parameter, L is the set of different lengths of action-state-reward triplets, W_l_ is the fraction of l-length triplets in the dataset, and finally ${\uprho }_{{\mathrm{T}}_{\mathrm{i}}}$ is the importance weight. This estimator allows for different length patient trajectories, which is critical in our case.

We utilize a simple bootstrap sample of 100 samples each and report the mean and 90% confidence interval of the overall risk estimate for each hypothetical treatment policy, defined in the next section.

3. Construction and Evaluation of Hypothetical Clinical Policies

We constructed four hypothetical treatment policies to evaluate to demonstrate the off-policy evaluation method. The first two treatment policies were based on the AST to Platelet Ratio Index (APRI). APRI is a validated predictor of hepatic fibrosis in chronic HCV routinely used in clinical care. Past work has used two APRI scores greater than 2.25 as a surrogate outcome for cirrhosis [16].

**Fig. 2:**
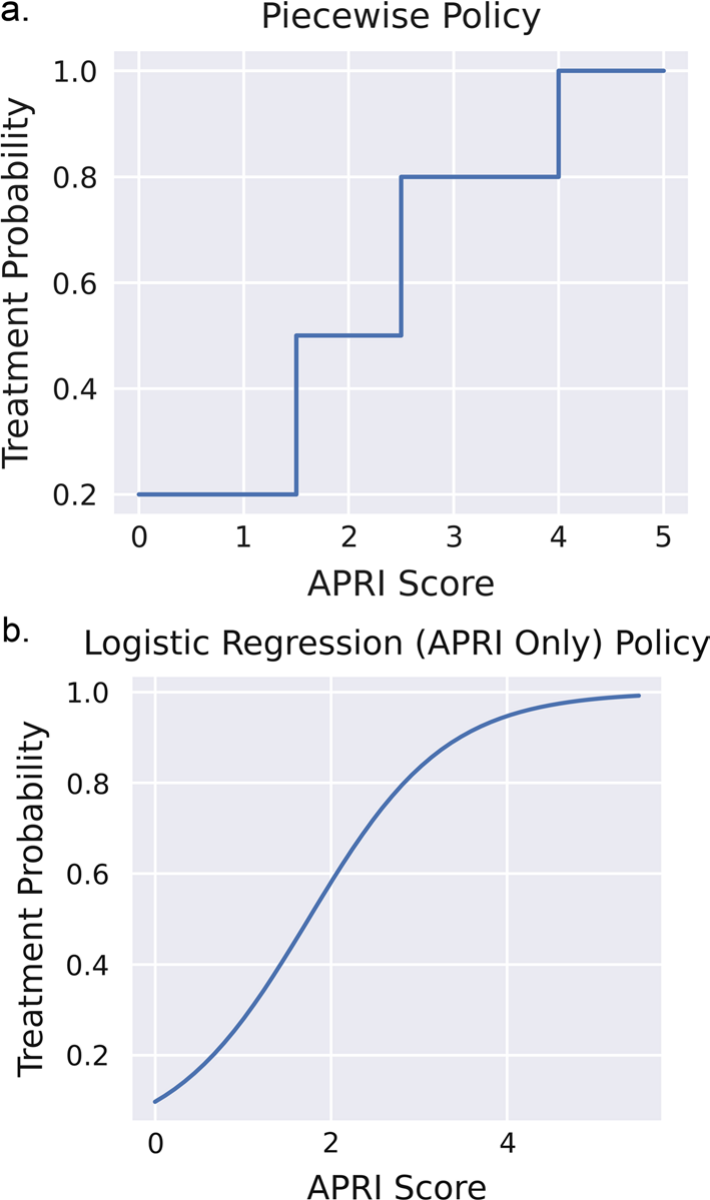
Treatment probabilities as a function of APRI score for the (**a**) piecewise treatment policy and (**b**) logistic regression (APRI only) policy. The first treatment policy is a piecewise function where treatment probability increases with APRI score (**a**). The second treatment policy is a data-driven treatment policy using logistic regression with APRI as a single feature, and the outcome being a positive diagnosis of cirrhosis (**b**)

*Policy 1—Piecewise Treatment Policy*: The first treatment policy is a piecewise function (Fig. 2a), where treatment probability increases with APRI score (Eq. 4):

4 $$P({\text{Treat}}|{\text{APRI}}) = \left\{ {\begin{array}{*{20}l} {0.2} \hfill & {{\text{APRI}} < 1.5} \hfill \\ {0.5} \hfill & {1.5 \le {\text{APRI}} < 2.5} \hfill \\ {0.8} \hfill & {2.5 \le {\text{APRI}} < 4} \hfill \\ {1.0} \hfill & {{\text{APRI}} > 4} \hfill \\ \end{array} } \right.$$

*Policy 2—Logistic Regression (APRI only)*: The second treatment policy is a data-driven treatment policy using logistic regression with APRI as a single feature. The outcome is a positive diagnosis of cirrhosis (Fig. 2b). The data points used to fit the logistic regression are the last APRI score recorded per patient before a diagnosis of cirrhosis (for positive outcome), or the last APRI score recorded for the patient (for negative outcome), taken from an expanded dataset where the outcome of cirrhosis was identified using liver elastography. The class probabilities are then used as treatment probabilities for the policy.

*Policy 3—Logistic Regression (All Variables)*: The third treatment policy is a logistic regression on all available state variables, with the same process as the previous treatment policy, and fit on the same expanded dataset as described above.

*Policy 4—Risk-Based Policy*: The final treatment policy is based on the risk measure used as the evaluator of patient risk. This policy is included to demonstrate the utility of incorporating each patient’s risk in the treatment decision. In particular, the probability of treatment ${\uppi }_{\mathrm{e}}(\mathrm{s})$ is based on a logistic function, i.e., ${\pi }_{e}\left(s\right)=\frac{{e}^{k(r\left(s\right)-{r}_{0})}}{1+{e}^{k(r\left(s\right)-{r}_{0})}}$ , where ${\uppi }_{\mathrm{e}}(\mathrm{s})$ is the probability of treatment given the state and $\mathrm{r}(\mathrm{s})$ is the calculated risk given the current state. For the policy in the results with the reported risk, ${\mathrm{r}}_{0}$ was set to ${\mathrm{r}}_{0}=0.003$, and k was set to 1000. These parameters were chosen as they were local minimal for the risk through a grid search. An investigation of how risk behaves as these parameters change is also explored in the results.

### Evaluation of treatment strategies using monte Carlo simulation

In addition to evaluating the estimated risk of each hypothetical policy, we also used a Monte Carlo simulation on the data to find the number of patients that the policies would treat. To avoid counterfactual evaluations, the Monte Carlo simulation is only performed on measurement time points for which the treatment has not started or has started at that time point. For each hypothetical treatment policy, we record the average number of patients treated, as well as the number of time points that were not treated. The latter can be thought of as one of many surrogates for cost savings, as delayed treatment implies a delayed expenditure for the hospital. Patients were separated into the low, medium, and high-risk categories by their maximum risk score overall measurement time points. Patients with a risk score above the 90th percentile (r = 0.015) were considered high-risk patients. Patients between the 90th percentile and 50th percentile (r = 0.0029) were considered medium risk, and those in the bottom half of the maximum risk scores were considered low risk. There were 380 high-risk patients, 1530 medium-risk patients, and 1882 low-risk patients used by the Monte Carlo simulation.

## Results

### Feature representation for actions, states, and risks

**Fig. 3:**
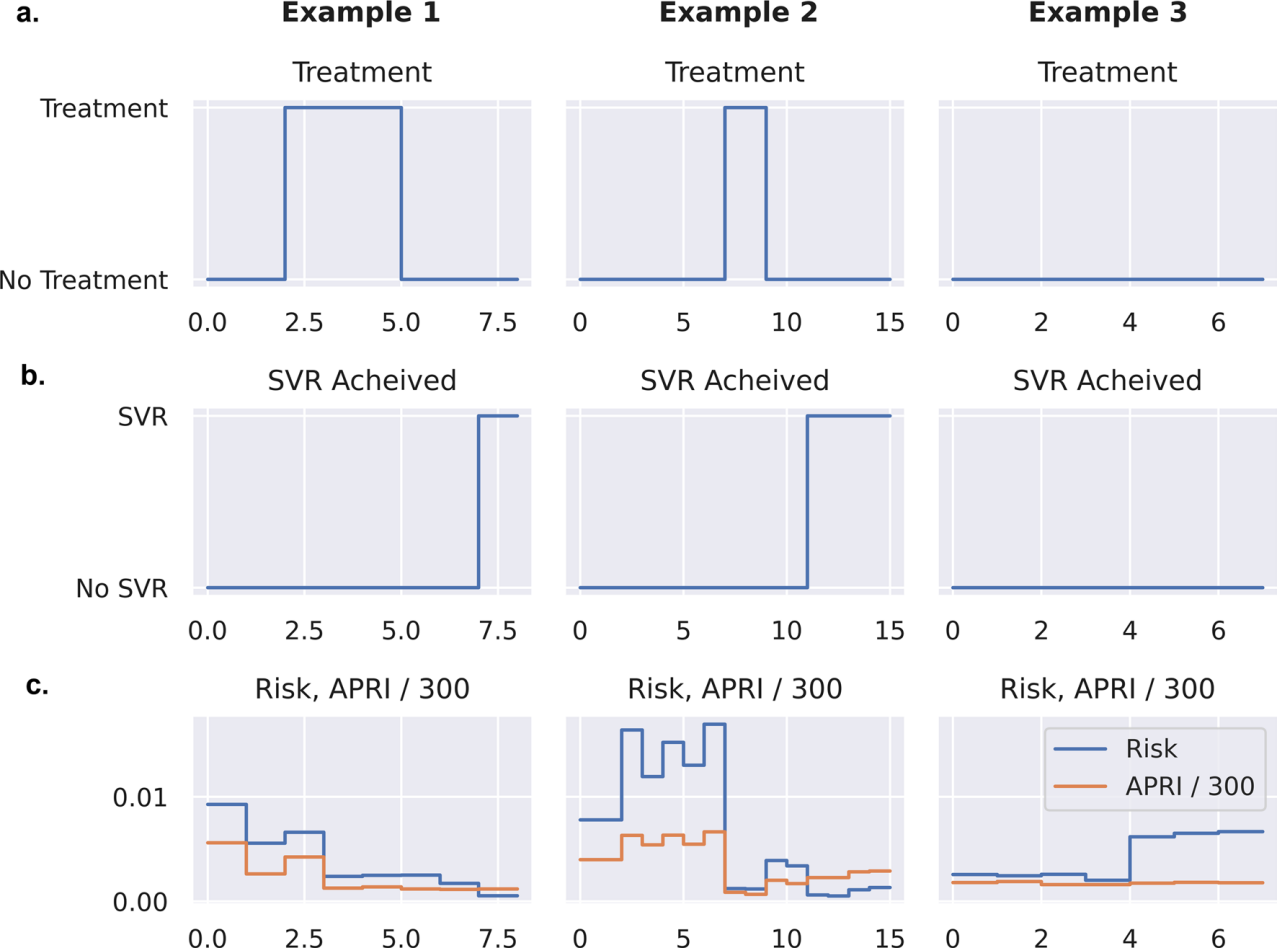
Three example traces for patients. Treatment decisions over time (**a**), SVR status (**b**), and risk score for development of cirrhosis and APRI/300, where 300 was chosen to place risk and APRI on similar scales (**c**) are displayed

**Fig. 4:**
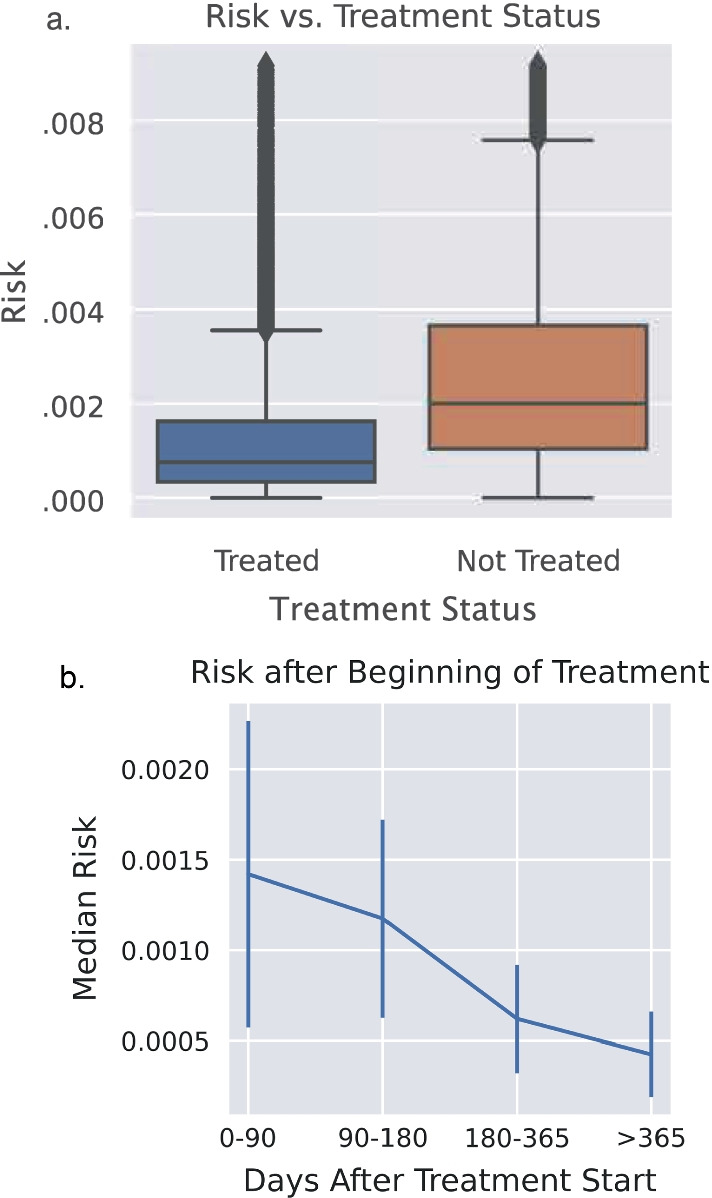
Analysis of risk scores. **a** Comparison of risk scores in the dataset separated between treated and untreated measurement timepoints. As expected, the untreated timepoints have a higher risk score on average. **b** Median risk (with 50% percentile interval) striated across amount of time after treatment start date. As expected, risk continues to decrease after initial treatment

Figure 3 shows three example traces for patients. Figure 3a shows treatment decisions over time. Figure 3b shows the associated drop in the risk of developing cirrhosis. Figure 3c shows an example of an untreated patient. Note that the risk of cirrhosis drops significantly as treatment starts; even before SVR is achieved, many patients’ longitudinal measurements (including APRI) improve rapidly after the start of treatment. This is evident in Fig. 4a, which compares the risk scores of treated vs. untreated patients; the median risk of treated patients is 69% lower than untreated patients (median 0.0007 vs. 0.0022, respectively). Note that the absolute scale of the risk is not important, rather the relative scale. Figure 4b shows the median risk as a function of the time after the treatment start date. As expected, the risk of developing cirrhosis decreases after treatment and continues to decrease after the end of DAA treatment.

### Off-policy evaluation and hypothetical clinical policies testing

Table 1 shows the results from the off-policy evaluation of the proposed policies. The risk-based policy has the lowest average risk of all the policies tested, with a bootstrap 90% confidence interval of (0.016, 0.019), with the full state logistic regression policy following behind. Policy 1 and Policy 2 are statistically nearly identical, as their treatment probability curves were shown to be similar in Fig. 2. Interestingly, although the risk-based policy has the lowest average risk of the tested policies, it also treats the least low-risk patients and the highest and medium-risk patients, showing that it can prioritize patients based on estimated risk. The nearest competitor, Policy 3, also has significantly fewer untreated time points, signaling that the risk-based policy is better at finding higher-risk patients at a lower cost to the health system.

**Table 1:** Expected risk of baseline and evaluation policies

	Risk score	90% Bootstrap CI	Number of patients treated	High risk (n = 380)	Medium risk (n = 1530)	Low risk (n = 1882)	Untreated timepoints
Policy 1: Piecewise Policy	0.028	(0.027, 0.033)	2018.5 + − 17.8	307.2 + − 6.9	893.9 + − 13.8	817.4 + − 15.7	7040.8 + − 127.5
Policy 2: Logistic Regression (APRI Only)	0.026	(0.024, 0.031)	1914.4 + − 18.6	316.2 + − 4.7	919.6 + − 16.7	672.3 + − 12.4	7742.9 + − 141.9
Policy 3: Logistic Regression (Full State)	0.023	(0.022, 0.029)	1637 + − 15.8	311.2 + − 5.8	850.2 + − 9.7	475.6 + _ 8.8	8877.4 + − 97.9
Policy 4: Risk-Based Policy	0.016	(0.016, 0.019)	1843.7 + − 16.5	346.4 + − 1.4	1121.7 + − 13.8	361.0 + − 20.1	7968.4 + − 110.4

**Fig. 5:**
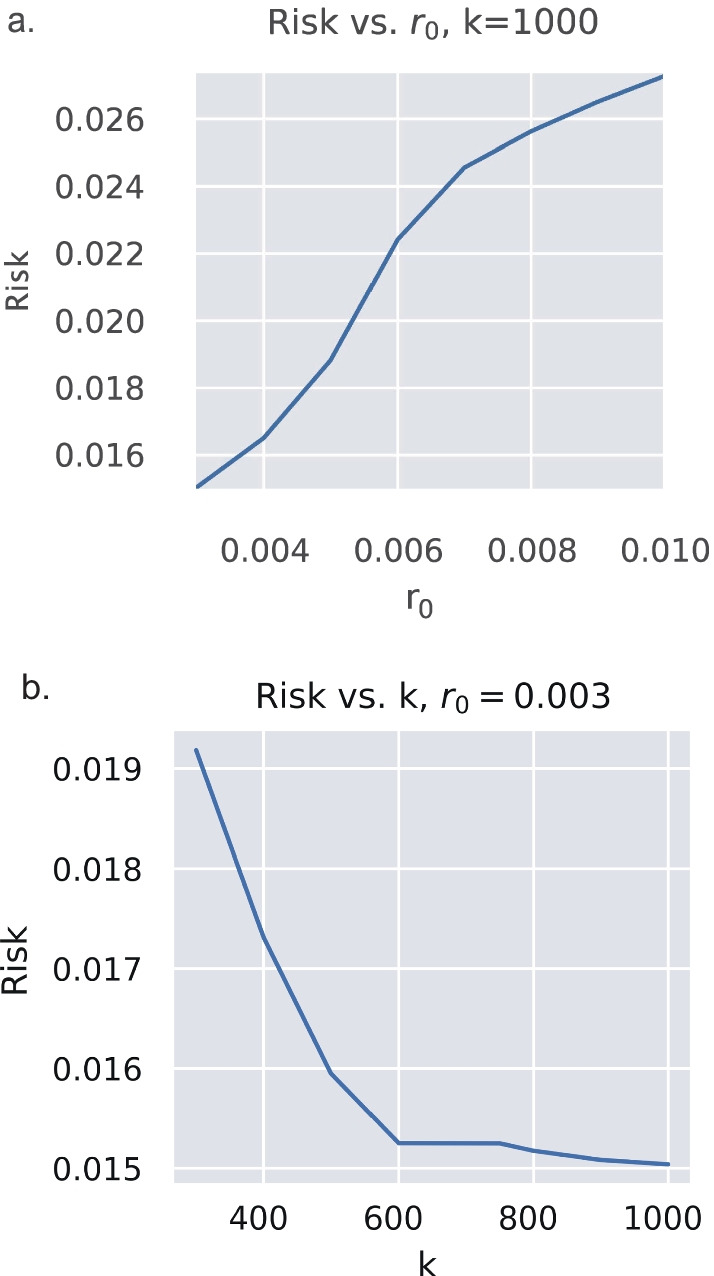
Risk sensitivity for k and ${r}_{0}$ . Sensitivity of the risk-based policy to ${\mathrm{r}}_{0}$ and$k$. As ${r}_{0}$ increases, risk also increases for a fixed $k$(**a**), and as $k$ increases, ${r}_{0}$ decreases but stabilizes for a fixed ${r}_{0}$(**b**)

Figure 5 shows the sensitivity of the risk-based policy to ${r}_{0}$ and $k$. As ${r}_{0}$ increases, risk also increases for a fixed $k$ (Fig. 5a), and as *k* increases, ${r}_{0}$ decreases but stabilizes for a fixed ${r}_{0}$ (Fig. 5b). Note that the Monte Carlo number of patients treated increases as risk decreases, and the number of untreated time points decreases with risk, as expected.

## Discussion

This study aimed to demonstrate the effectiveness of a reinforcement learning framework for the evaluation of hypothetical treatment policies using historical data. Through the case study of HCV treatment, we demonstrated that off-policy evaluation could be helpful to compare different intervention strategies in advance. Using this approach, we found that a risk-based policy had the best estimated average risk score and thus best prioritized treating medium- and high-risk patients over low-risk patients compared to other hypothetical treatment policies. The clinical implication in implementing a risk-based policy is more efficient treatment allocation. Therefore, this approach could have helped prioritize short-term access and costs but also could have helped save dollars to avert downstream cirrhosis-related management and complications for large health systems.

This type of evaluation approach can be helpful for other disease states where similar policy comparisons are needed and can be used as a generalized methodological tool for evaluating treatment strategies without waiting for outcomes from a clinical study. For example, this can be applied to other diseases with high-cost conditions such as cancers [23].

These results also imply that, under financial limitations or treatment scarcity, systematic treatment policies could improve average patient outcomes, as measured by a reduced risk in cirrhosis. While outside of the scope of this paper, we believe that more complicated policies could further improve the average outcome without requiring immediate treatment of all enrolled patients. Indeed, for HCV, more complex classification models could also be extended to create new treatment policies.

While the technique of off-policy evaluation is only applied in this study to treatment policies for HCV, the methodology is applicable in many different scenarios. The key ingredients necessary for policy evaluation here are a predefined risk/reward measurement or model, a well-defined action space, and a series of states. The careful construction of these components is essential, as different design decisions regarding the construction of these variables will significantly affect the overall outcome. Other work has considered these techniques in the cases of sepsis treatment [21], and more applications of this type are sure to follow. Although simulation modeling can be used to evaluate theoretical decision paths in advance, they are based on assumptions that may fail to provide unbiased evaluations of the hypothetical treatment policy [7]. Future studies will focus on the clinical validation of these results.

## Limitations

This approach has several important limitations; first, the methodology assumes that the treatment action is based solely on the state at that particular time and not the past trajectory of the patient. This is not how clinicians operate in practice, although we find it a reasonable approximation here. The second limitation is that missing data were imputed by using the last observation carried forward. Other potential imputation methods could have been utilized that produce less bias in the final policy estimates. Both the baseline and each of the hypothetical treatment policies are assumed to be randomized, i.e., the treatment decisions made at a particular time point are stochastic. This can prove troublesome in implementing these policies in a real-world environment, where hard treatment thresholds are usually favored. Some adjustments can allow for deterministic policies, but this generally requires more data to represent the joint state-action space properly; we leave this to future work. Another important limitation of this method is that although we reported treatment quantity, this work does not explicitly allow for a budget constraint on the amount of treatment given, either in total or over time. An important additional limitation in this work concerns the robustness and generalizability of the technique. From a methodological perspective, a more sophisticated means of determining the parameters would be better to ensure the optimality of the policy. A final limitation of this study is that patients treated with DAAs but do not achieve SVR are excluded from this study. Although this is a small population of treated patients, our current technique cannot account for this outcome. In addition, we note that a more generalizable and externally validated risk model would give the method presented in this paper more empirical credibility to inform policy decisions.

## Conclusion

New hypothetical treatment policies for HCV were evaluated using a reinforcement learning framework using historical data collected from the VHA. A risk-based policy was shown to prioritize high and medium-risk patients more effectively while reducing the cost to healthcare systems. The methodology used in this study could be of interest for a better understanding of treatment policies for other diseases.

## Supplementary Information

**Additional file 1**. Coefficients for Time-varying Cox Model for Cirrhosis at 1 year.

## Data Availability

These analyses were performed using data from the Corporate Warehouse Domains that are available only within the US Department of Veterans Affairs firewall in a secure research environment, the VA Informatics and Computing Infrastructure (VINCI). In order to comply with VA privacy and data security policies and regulatory constraints, only aggregate summary statistics and results of our analyses are permitted to be removed from the data warehouse for publication. The authors have provided detailed results of the analyses in the paper. These restrictions are in place in order to maintain Veteran privacy and confidentiality. Access to these data can be granted to persons who are not employees of the VA; however, there is an official protocol that must be followed for doing so. The authors also confirm that VA policies are currently being developed that should allow an interested researcher to obtain a de-identified, raw dataset upon request with a data use agreement. Those wishing to access the data that were used for this analysis may contact Jennifer Burns, MHSA, who is a senior data manager at the VA Center for Clinical Management Research, to discuss the details of the VA data access approval process. Her contact information is as follows: Email: Jennifer.Burns@va.gov. UM North Campus Research Complex, Department of Veterans Affairs, 2800 Plymouth Road Bldg 16, Ann Arbor, MI. She has access to the data, knowledge of the current and evolving VA policies for sharing data, and is included on the IRB for this study. The corresponding author, Akbar Waljee, MD (awaljee@med.umich.edu) is also available to discuss the details of the VA data access and approval process. The code used in this study has been uploaded to the following GitHub link: Github.com/CCMRcodes/HCV-policy.

## Abbreviations

APRI: Aspartate Aminotransferase to Platelet Ratio Index
AST: Aspartate Aminotransferase
ALT: Alanine Aminotransferase
DAA: Direct-Acting Antivirals
SVR: Sustained Virologic Response
VHA: Veterans Health Administration
