# Supplementary material for: Reinforcement learning evaluation of treatment policies for patients with hepatitis C virus
Source: BMC Med Inform Decis Mak. 2022 Mar 11;22:63. doi: 10.1186/s12911-022-01789-7 (PMC8913329; doi:10.1186/s12911-022-01789-7)
Supplement: Supplementary file 1 — Additional file 1. Coefficients for Time-varying Cox Model for Cirrhosis at 1 year. [file 12911_2022_1789_MOESM1_ESM.docx]

**SUPPLEMENTARY MATERIALS**

**SUPPLEMENTAL TABLES**

| **Supplementary Table 1**. Coefficients for Time-varying Cox Model for Cirrhosis at 1 year. | | | | |
| --- | --- | --- | --- | --- |
|  | coefficient | standard error (coeff) | z | p-value |
| Albumin | -0.5461 | 0.0157 | -34.8 | <.001 |
| Alkaline phosphatase | 0.1582 | 0.009 | 17.53 | <.001 |
| ALT | 0.0307 | 0.004 | 7.652 | <.001 |
| AST | 0.5346 | 0.0071 | 75.529 | <.001 |
| Total bilirubin | 0.0849 | 0.0108 | 7.841 | <.001 |
| BUN | -0.0064 | 0.0011 | -5.714 | <.001 |
| Chloride | -0.0041 | 0.0023 | -1.757 | 0.08 |
| Creatinine | 0.0157 | 0.0085 | 1.976 | 0.48 |
| Glucose | -0.0001 | 0.0001 | 0.94 | 0.35 |
| Hemoglobin | -0.024 | 0.0044 | -5.458 | <.001 |
| Platelets | -0.0116 | 0.0001 | -83.162 | <.001 |
| Potassium | -0.0224 | 0.0158 | -1.418 | 0.16 |
| Sodium | -0.0151 | 0.0028 | -5.418 | <.001 |
| White blood cell count | 0.0179 | 0.0031 | 5.879 | <.001 |
| APRI | -0.0043 | 0.0069 | -0.618 | <.001 |
| AST: ALT ratio | 0.1069 | 0.0069 | 15.594 | <.001 |
| SVR | -1.3737 | 0.101 | -13.6 | <.001 |
| Age at first APRI | -0.0069 | 0.0009 | -7.544 | <.001 |
| Hispanic race | 0.4054 | 0.0301 | 13.446 | <.001 |
| Missing race | 0.3397 | 0.029 | 11.729 | <.001 |
| Other race | 0.2522 | 0.0507 | 4.973 | <.001 |
| White race | 0.3264 | 0.0156 | 20.877 | <.001 |
| Male sex | 0.1145 | 0.0447 | 2.561 | 0.01 |
